# Market surveys and social media provide confirmation of the endangered giant freshwater whipray *Urogymnus polylepis* in Myanmar

**DOI:** 10.1111/jfb.15073

**Published:** 2022-05-06

**Authors:** Michael I. Grant, Anthony W. J. Bicknell, Thaung Htut, Antt Maung, Thu Maung, Khin Myo Myo, Thu Rein, Min Khan San, William T. White, Kyaw Zay Ya, Meira Mizrahi

**Affiliations:** ^1^ CENTRE for Sustainable Tropical Fisheries and Aquaculture, College of Science and Engineering James Cook University Townsville Queensland Australia; ^2^ University of Exeter College of Life and Environmental Sciences Exeter UK; ^3^ Wildlife Conservation Society, Myanmar Programme Yangon Myanmar; ^4^ CSIRO Oceans and Atmosphere Hobart Tasmania Australia; ^5^ Australian National Fish Collection CSIRO National Research Collections Australia Hobart Tasmania Australia

**Keywords:** biodiversity, Chin State, Facebook, non‐marine elasmobranchs, Rakhine, threatened species

## Abstract

The giant freshwater whipray *Urogymnus polylepis* is a threatened species that is vulnerable to riverine and coastal marine pressures. Despite its threatened status, the range of *U*. *polylepis* is still being determined. In this study, photographic evidence of *U*. *polylepis* in Myanmar was provided through market surveys (2017–2018) and social media (Sharks and Rays of Rakhine Facebook page, 2021). *Urogymnus polylepis* is exposed to fisheries and habitat degradation pressures in Myanmar; therefore, conservation management is likely needed to ensure populations persist into the future.

There is global concern about the deterioration of riverine environments. In the tropics, riverine environments have degraded through a range of human‐induced activities, such as the construction of water retention structures (Grill *et al*., 2019); general land repurposing for agriculture; and residential, commercial and industrial development within catchments (Vörösmarty *et al*., 2010). These activities have collectively altered, fragmented and reduced the quality of available riverine habitat. Intensive inland fisheries (*e*.*g*., Ainsworth *et al*., 2021; Funge‐Smith, 2018) and species introductions have additionally compounded these pressures, resulting in a freshwater crisis (Su *et al*., 2021; Tickner *et al*., 2020), with large fish species being particularly affected (He *et al*., 2019).

Among freshwater fishes, there is very little information on the conservation status of non‐marine elasmobranchs (Grant *et al*., 2019). Elasmobranchs that use freshwater environments are either freshwater obligates (45 described species, Grant *et al*., 2019; Loboda *et al*., 2021) or euryhaline generalists (10 species), which use a range of freshwater, estuarine and marine habitats throughout their life history (Grant *et al*., 2019). In the Indo‐Pacific, most non‐marine elasmobranchs are threatened with extinction on the IUCN Red List of Threatened Species (hereafter IUCN Red List, IUCN. 2021). These species are, however, poorly studied, and fundamental information is still required on aspects of their biology and distribution.

The giant freshwater whipray *Urogymnus polylepis* is one of the largest ray species, attaining sizes of at least 223 cm disc width (Grant *et al*., 2021b). It is a euryhaline species with fragmented populations across Indonesia (Java, Kalimantan and Sumatra), Malaysia (Peninsular and Borneo), Brunei, the Mekong River basin (including Laos, Thailand, Vietnam and Cambodia), Thailand, Bangladesh and India (Grant *et al*., 2021b). Within this range, *U*. *polylepis* is exposed to intense small‐scale (subsistence and artisanal) fisheries (*e*.*g*., Funge‐Smith, 2018; Haque *et al*., 2021), as well as targeted recreational fisheries and harvest for ornamental aquaculture (*e*.*g*., Compagno & Cook, 2005).

Despite the charismatically large size of *U*. *polylepis*, its global range is still being determined. It is only recently that this species has been observed broadly throughout the island of Borneo (Windusari *et al*., 2019), Indonesia (Iqbal *et al*., 2020) and Peninsular Malaysia (Iqbal *et al*., 2019), and contemporary observations in India (Ishihara *et al*., 1998; Sen *et al*., 2020) and Bangladesh (Haque *et al*., 2021) have been limited following the historic description of the junior synonym *Trygon fluviatilis* (Annandale, 1910) from the lower Ganges River. Although conspecific subpopulations across this range are presently considered to be *U*. *polylepis*, the possibility of some subpopulations being separate species remains. For example, large divergences in sequences of the cytochrome *b* gene have been found between populations in India and Thailand (Sezaki *et al*., 1999).

A persistent ambiguity within the range of *U*. *polylepis* has been whether it occurs in Myanmar (formerly Burma). Vidthayanon *et al*. (2013) had mentioned possible reports of this species from Myanmar, whereas Grant *et al*. (2021b) recently considered *U*. *polylepis* as “possibly extant” in the lower Ayeyarwady basin based on Vidthayanon *et al*. (2013) and because the Ayeyarwady basin presents an ideal expanse of riverine habitat situated between populations in the Sundarbans‐Ganges River and Southeast Asia. Furthermore, a potential range of *U. polylepis* also lies in rivers of northern Rakhine, which has been subject to ongoing civil insurgency since the 1950s. Nonetheless, the presence of *U*. *polylepis* in Myanmar has not yet been verified. This is partly because field studies in Myanmar are logistically challenging to conduct and further complicated by regular political unrest in recent decades.

The aim of this paper is to provide photographic evidence of *U*. *polylepis* in Rakhine and Chin State, Myanmar, that resulted from landing site surveys and social media data collection. Landing site surveys were conducted over three seasons (rainy, cool and dry) between 2017 and 2018. These surveys aimed to obtain baseline biological information on shark and ray species in Rakhine State (*i*.*e*., presence, diversity, spatial distribution, sex and size and gear susceptibility). The authors followed the survey protocol outlined in the Wildlife Conservation Society (WCS) Field Manual for Shark and Ray Fisher, Trader & Market Based Surveys in Rakhine, Myanmar (Bicknel, 2017). All surveys were conducted in Myanmar language by native Myanmar speakers. At each landing site, the authors identified boats that had shark or ray landings and obtained verbal consent from the fishers to conduct the survey. Before recording any landings data, the authors obtained information related to boat details, gear type, target species and fishing activity. For each ray specimen, they recorded the species, disc length, disc width, weight and sex. They also took a photograph of each specimen to verify species identifications. From these market surveys, two records of *U*. *polylepis* were made from Sittwe Market in July and December 2017 (Figure 1; Table 1).

**FIGURE 1:**
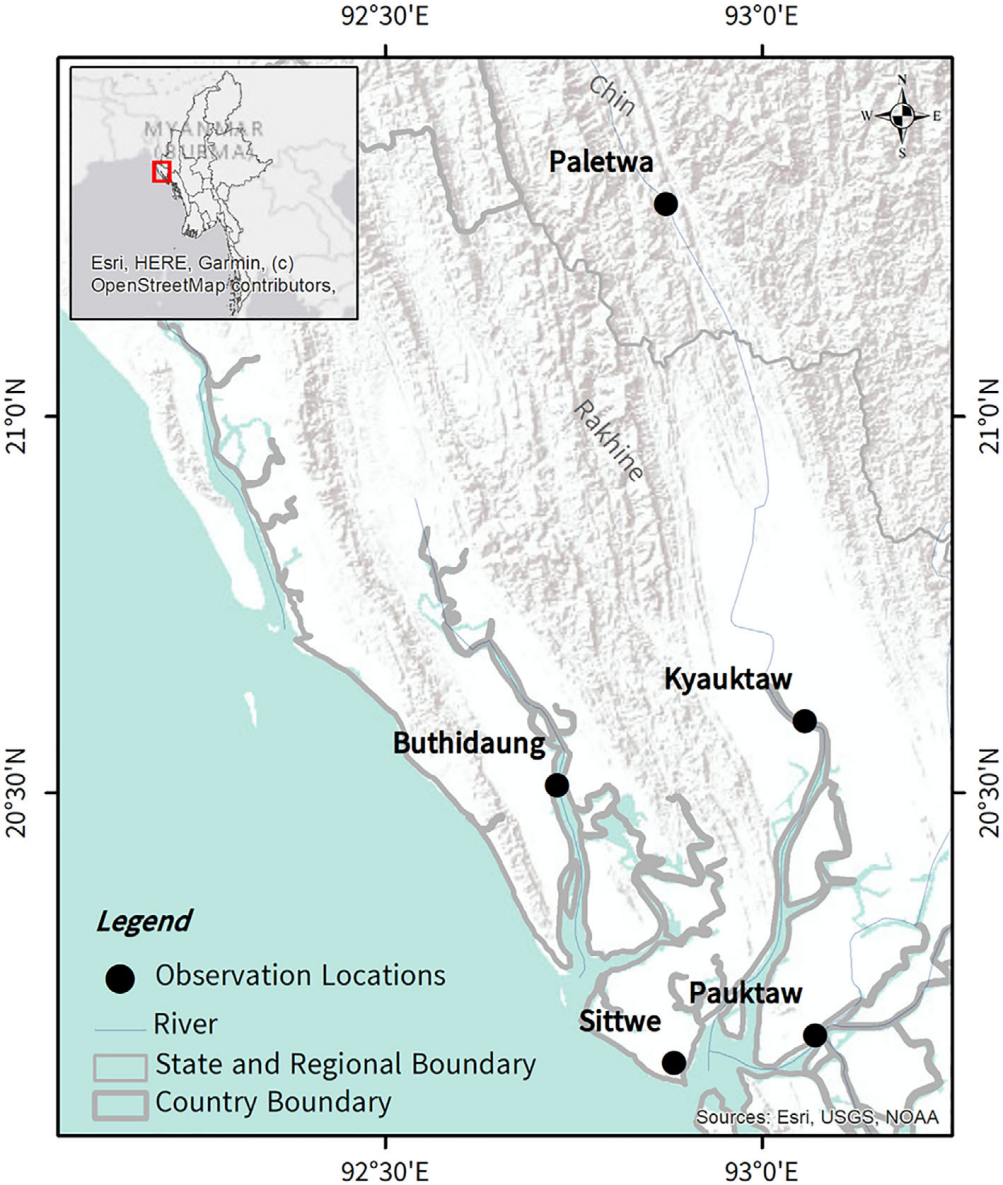
Distribution of *Urogymnus polylepis* observations in Myanmar.

**TABLE 1:** Available information for records of *Urogymnus polylepis* in Rakhine and Chin States, Myanmar

Observation method	Date of capture	River system	State/region	Town/location	Coordinates	Gear type	Comments	Figure 2 reference
Facebook	May 2016	Kaladan River	Rakhine	Kyauktaw	20° 35′ 44.980″ N 93° 3′ 27.990″ E	Longline		E–F
Facebook	25 September 2016	Mayu River	Rakhine	Buthidaung	20° 30′ 38.150″ N 92° 43′ 42.560″ E	Longline		H
Facebook	May 2017	Mayu River	Rakhine	Buthidaung	20° 30′ 38.150″ N 92° 43′ 42.560″ E	Longline		G
Market surveys	July 2017	Kaladan River	Rakhine	Sittwe	20° 8′ 29.040″ N 92° 53′ 0.180″ E	Longline	Female, DL = 129 cm, DW = 122 cm, weight = *c*. 130 kg, price = 210,000 MMK (=US$118.2, January 2021)	I
Market surveys	21 December 2017	Kaladan River	Rakhine	Sittwe	20° 8′ 29.040″ N 92° 53′ 0.180″ E	Tidal net/fence net	Female, DL = 194 cm, DW = 178 cm	J–K
Facebook	April 2018	Kaladan River	Rakhine	Pauktaw	20° 10′ 41.963″ N 93° 4′ 17.260″ E	Longline		A–D
Facebook	June 2021	Kaladan River	Chin	Kinwa, Paletwa	21° 16′ 58.314″ N 92° 52′ 22.703″ E	Longline	Female; birthed two pups upon capture	L–M

*Note*. DL, disc length; DW, disc width; MMK, Myanmar Kyat.

A post was additionally made on the Sharks and Rays of Rakhine Facebook group (64 members) on 9 June 2021, where users were invited to share any photographs of large stingrays caught locally within riverine environments. An additional five records were received from Facebook users, and consent for use of their images in publication was received. These records were dated between 2016 and 2021 from fishing communities along the Mayu and Kalatan Rivers in Rakhine and Chin States (Figure 1).

In sum, photographs of six specimens and one video of a large female with two pups were obtained from five locations (Figure 1; Table 1). Although the quality of photographic evidence varied, each of the specimens pictured (Figure 2) were clearly very large, uniformly brown stingrays. The broadly oval‐to‐subcircular disc, broad snout with an enlarged narrow apical lobe and minute eyes are distinctive of *U*. *polylepis* (see Last *et al*., 2016). Five of these specimens were reportedly caught in riverine environments, but no catch location was available for the Sittwe Market observations. One pregnant female was observed in an upstream freshwater environment at Kinwa, Paletwa, in the Kaladan River. This female pupped two well‐developed neonates upon capture (Figure 2l,m), supporting suggestions that parturition and nursery areas occur in freshwater environments for this species (Grant *et al*., 2019).

**FIGURE 2:**
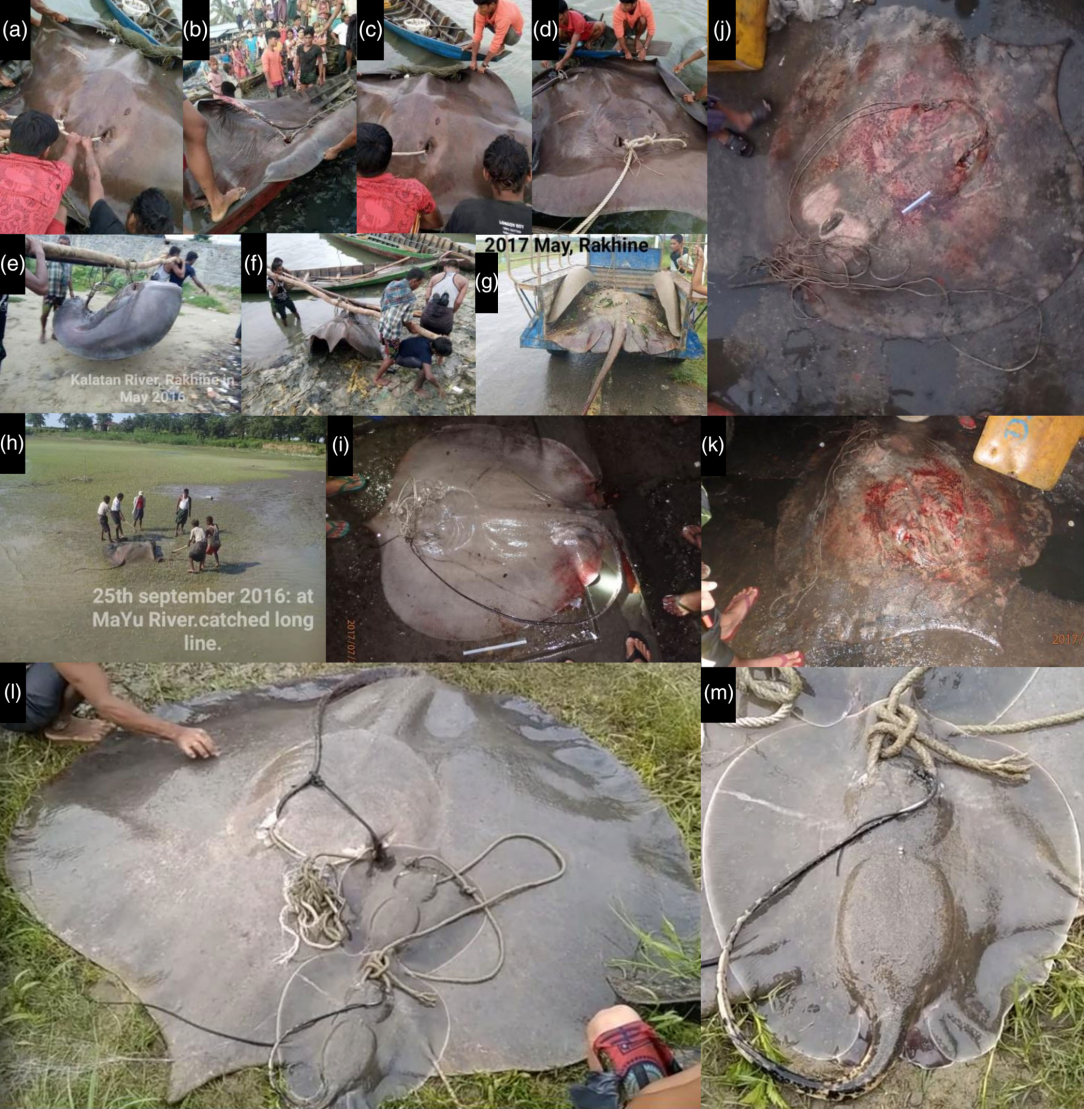
Photographs of *Urogymnus polylepis* in Rakhine and Chin States, Myanmar. (a–d) Pauktaw, (e, f) Kyautaw, (g, h) Buthidaung, (i–k) Sittwe Market and (l, m) Kinwa Paletwa (Table 1)

The two neonates observed provide new information on the morphology of *U*. *polylepis*. The dorsal disc of both neonates had a narrow white margin and two or three enlarged denticles on the scapular region (Figure 2l,m). Both these features have not previously been noted for *U*. *polylepis* and are absent in larger specimens (*e*.*g*., Last *et al*., 2016), including those observed in the current study. This indicates that these features are possibly unique to neonates. Collectively, these morphological features indicate the potential for misidentification with the white‐edge whipray *Fluvitrygon signifer*, an obligate freshwater species with an overlapping distribution in rivers of Southeast Asia (Grant *et al*., 2019). *F. signifer* also has a ventral white margin on its disc and a pearl spine on the scapular region as juveniles (Compagno & Roberts, 1982; Last *et al*., 2016). The tail provides a distinguishing characteristic between neonate *U*. *polylepis* and *F*. *signifer*: *U*. *polylepis* has a dark‐coloured tail, covered entirely in denticles, whereas *F*. *signifer* has a characteristically light‐coloured tail that is sparsely covered in denticles beyond the caudal stings (Last *et al*., 2016).

The observations of *U*. *polylepis* in the present study provide an important update on the range of this endangered species; nonetheless, there is concern about the status of local populations. Myanmar supports one of the world's largest per‐capita (24.46 kg per year) inland fisheries (Funge‐Smith, 2018), accounting for one‐third of Myanmar's total fisheries production (Baran *et al*., 2018). A diverse range of gear types are documented in Myanmar's inland fishery that includes various gillnet, hook, trap and electrofishing techniques (Baran *et al*., 2018; Soe *et al*., 2020). Whereas several studies have focused on inland fisheries catch in Myanmar (*e*.*g*., Baran *et al*., 2018; Lwin, 2017; Soe *et al*., 2020), reports of rays are, to the best of authors’ knowledge, either absent or unpublished. Marine rays are targeted heavily in coastal Rakhine, often dried for local consumption (WCS Myanmar, unpubl. data); therefore, it is possible that the same could be true for rays captured in inland fisheries. It is unclear whether the dearth in ray catch data in inland fisheries is due to large historic depletions before detailed catch landing surveys were conducted, if local gear is ineffective in capturing rays, or simply due to the focus of these studies towards other taxa. A further reason may be due to the subsistence nature (*i*.*e*., fishing directly for household consumption) of inland fisheries in Myanmar, limiting the amount of catch that is sold in markets and thus more easily observable. Apart from fishing, riverine environments in Myanmar have been significantly degraded by land repurposing activities and potentially from mining pollutants (Grant *et al*., 2021a). In particular, deforestation of mangroves in the lower delta estuarine areas of the Ayeyarwady River and Rakhine State has increased dramatically in recent decades (Estoque *et al*., 2018).

Only four other non‐marine elasmobranch species are known from Myanmar. The Chindwin cowtail ray *Makararaja chindwinensis* is known from only two recorded specimens in the Chindwin River tributary of the Ayeyarwady basin (Grant *et al*., 2021a); the Ganges River shark *Glyphis gangeticus* has not been observed in Myanmar since the description of the junior synonym *Glyphis* (*Prionodon*) *siamensis* in the late 19th century (Li *et al*., 2015); the largetooth sawfish *Pristis pristis* has limited available records in Myanmar, and its presence is considered “uncertain” (Dulvy *et al*., 2016), whereas only the bull shark *Carcharhinus leucas* is still regularly observed in marine catch landings (*e*.*g*., Howard *et al*., 2015). Considering the conservation status of these other non‐marine elasmobranch species in Myanmar, the present range extension of *U*. *polylepis* is unlikely to provide a globally significant refuge for this species, as populations are exposed to a combination of fisheries and habitat degradation pressures.

*U. polylepis* likely requires a concerted conservation effort in Myanmar to ensure populations persist into the future. Furthermore, the presence of *U*. *polylepis* in the Ayeyarwady basin still requires verification. With the present political unrest in Myanmar, the use of social media in the present study has provided an effective tool to document the distribution of a distinctive and poorly known threatened species and may have further applications for conservation of elasmobranchs in Myanmar (Di Minin *et al*., 2015). Use of social media platforms to generate citizen science and track public perception of protected species (*e*.*g*., Kroetz *et al*., 2021) and collect data for cryptic and poorly known species (*e*.*g*., McDavitt & Kyne, 2020), or from regions that are logistically difficult to conduct surveys in (Iqbal *et al*., 2017, Iqbal *et al*., 2018), has been successfully applied to threatened elasmobranchs. It is likely that social media can provide further information on Myanmar's data‐poor elasmobranch species into the future (*e*.*g*., *M*. *chindwinensis*) and continue to have broad applications to conservation of elasmobranchs globally.

## AUTHOR CONTRIBUTIONS

M.I.G. and M.M. were involved in conception of this study; A.B., T.H., A.M., K.M.M., T.R., M.K.S. and M.M. helped in data generation; M.I.G., T.H., A.M., T.M., K.M.M., T.R., M.K.S., W.T.W., K.Z.Y. and M.M. assisted with data analysis; M.I.G and M.M. were involved in manuscript preparation; all other authors contributed to editing the manuscript.
